# Relationship Between Hospital Team Segregation Index, Heart Failure, and Ischemic Heart Disease

**DOI:** 10.1016/j.jacadv.2025.101988

**Published:** 2025-07-18

**Authors:** Adedoyin Johnson, Shannon M. Knapp, Hunter Mwansa, Janina Quintero Bisonó, Ikeoluwapo Kendra Bolakale-Rufai, Wanda Moore, Ekow Yankah, Ryan Yee, Dalancee Trabue, Francesca Williamson, Natalie Pool, Megan Hebdon, Quinn Capers, Courtland Blount, Nia Kimbrough, Denee Johnson, Jalynn Evans, Brandi Foree, Rev Anastasia Holman, David Brown, Brownsyne Tucker Edmonds, Brahmajee Nallamothu, John Hollingsworth, Khadijah Breathett

**Affiliations:** a Department of Internal Medicine, Indiana University, Indianapolis, Indiana, USA; b Division of Cardiovascular Medicine, Krannert Cardiovascular Research Center, Indiana University, Indianapolis, Indiana, USA; c Department of Biostatistics and Health Data Science, Richard M. Fairbanks School of Public Health, Indianapolis, Indiana, USA; d Sarver Heart Center Minority Outreach Program, University of Arizona, Tucson, Arizona, USA; e Department of Law and Philosophy, University of Michigan, Ann Arbor, Michigan, USA; f Department of Learning Health Sciences, University of Michigan, Ann Arbor, Michigan, USA; g School of Nursing, University of Northern Colorado, Greeley, Colorado, USA; h School of Nursing, University of Utah, Salt Lake City, Utah, USA; i Department of Medicine, Howard University Medical Center, Washington, District of Columbia, USA; j Centers of Wellness for Urban Women, Indianapolis, Indiana, USA; k Indiana University Health, Indianapolis, Indiana, USA; l Division of Chaplaincy Education, Indiana University Health System, Indianapolis, Indiana, USA; m Department of Otolaryngology-Head and Neck Surgery and Pediatric Otolaryngology, University of Michigan, Ann Arbor, Michigan, USA; n Department of Obstetrics and Gynecology, Indiana University, Indianapolis, Indiana, USA; o Division of Cardiovascular Diseases, University of Michigan, Ann Arbor, Michigan, USA; p Quality Department, Endeavor Health NorthShore Hospitals, Evanston, Illinois, USA

**Keywords:** cardiovascular outcomes, coronary heart disease, heart failure, ischemic heart disease, racial disparities

## Abstract

**Background:**

The extent of structural racism in cardiovascular care is not well studied.

**Objectives:**

The purpose of this study was to determine whether hospital segregation index (SI) (degree of dissimilarity between teams caring for Black vs White patients) is associated with receipt of a cardiologist’s care, readmission up to 30 days, and 1-year survival for Black vs White patients admitted with heart failure (HF) or ischemic heart disease (IHD).

**Methods:**

Using Optum’s Clinformatics Data Mart, we examined the effect of hospital SI on Black and White patients admitted with primary diagnoses of HF or IHD from 2010 to 2018 using mixed effects models. Hospitals were grouped by SI tertile.

**Results:**

Overall, 119,272 patients (29.4% Black, 70.6% White) with HF and 183,165 patients (17.9% Black and 82.1% White) with IHD were analyzed. Across SI tertiles, Black patients with HF had lower odds of receiving a cardiologist’s care compared to White patients. Across SI tertiles and cardiology care, there was no difference in the hazard of readmission by race, and odds of 1-year survival were higher for Black patients. Among patients with IHD, there was no difference in odds of receiving cardiology care between races across SI tertiles. Black patients not seen by cardiologists had 20% higher hazard of readmission at high SI hospitals (HR: 1.20; 95% CI: 1.00-1.44). Odds of 1-year survival were higher for Black patients (OR: 1.10; 95% CI: 1.04-1.17) seen by cardiologists in low SI hospitals.

**Conclusions:**

Among Black vs White patients, HF outcomes did not vary by SI tertiles. However, in IHD, SI impacted Black patients’ risk of readmission and survival.

Heart failure (HF) and ischemic heart disease (IHD) are major drivers of cardiovascular disease (CVD) attributable to morbidity and mortality in the United States.^1^ HF, a common sequela of IHD, accounts for 9.2% of CVD deaths and is projected to cost $69.8 billion by 2030.^1^^,^^2^ When adjusted for sex and social determinants of health (SDOH), the prevalence of IHD is similar in non-Hispanic (NH) Black and NH White individuals, but IHD outcomes (incident HF, recurrent myocardial infarction, angina) and mortality are worse in NH Black than NH White individuals.^3^^,^^4^ IHD age-adjusted mortality rate per 100,000 is 153.6 and 85.9 for NH Black male and female vs 128.5 and 63.8 for NH White male and female, respectively.^1^^,^^4^ NH Black, compared to NH White individuals, have a two-fold higher risk for incident HF, higher HF rehospitalization rates, and are more likely to die within 5 years of hospitalization for incident HF.^5^, ^6^, ^7^

Factors that contribute to racial disparities in HF and IHD outcomes are multifactorial, from physician-patient-level factors (eg, clinical inertia, comorbidities, implicit bias, health literacy, physician/patient racial/sex concordance, etc) to system-level factors such as hospital resources and structural racism.^8^^,^^9^ SDOH are at the intersection of physician/patient level and system factors and are responsible for over 50% of health outcomes.^10^^,^^11^ An important SDOH, access to health care delivery, requires further investigation. Specifically, the extent of structural racism is unclear in the delivery of inpatient cardiovascular care.

Hospital segregation index (SI) is an understudied metric for structural racism in health care delivery. SI measures the degree of dissimilarity in health care teams for patients in one group compared to patients in another group by hospital.^12^ We used Optum’s deidentified Clinformatics Data Mart (CDM) Database to examine SI, degree of dissimilarity, between health care teams that care for NH Black vs NH White patients with a primary diagnosis of HF or IHD. We evaluated hospital SI and its association with receipt of care by a cardiologist, time to readmission (up to 30 days), and 1-year survival for Black compared to White patients.

## Methods

### Data source

Optum’s deidentified CDM Database is a Health Insurance Portability and Accountability Act compliant system of Medicare Advantage and commercial health claims that includes approximately 67 million beneficiaries across the United States. This deidentified database includes patient demographics such as age, race (administratively defined by Optum), ethnicity, pharmacy claims, inpatient claims, and medical claims. Indiana University has a license with Optum to use their data for research purposes.^13^ The Indiana Institutional Review Board deemed this study exempt from review due to the deidentified data.

### Hospital SI

SI was estimated for hospitals using all admissions from 2010 to 2018 with a primary diagnosis of HF or IHD; SI for each diagnosis was calculated separately. Patients with race other than NH White (White) or NH Black (Black) and those with race unknown were excluded to initially focus on the population with greatest disparities. We only included hospitals with at least 10 White patients and at least 10 Black patients over the study period.

SI for a hospital was calculated following Hollingsworth et al 2021: 0.5∑|*b*_*i*_−*w*_*i*_|, where b_i_ is the relative frequency of Black patients for the ith provider in a hospital’s care team network and w_i_ is the relative frequency of White patients for the ith provider in a hospital’s care team network.^12^ The sum is over all providers in a hospital. SI ranges from zero to one. When SI equals zero, then each provider has the same proportion of Black and White patients as all other providers, but when SI equals one, the providers are completely separated treating only Black or White patients. Providers in Optum’s CDM were defined as any individual that comprised the medical team such as physicians, advanced practice providers, nurses, social workers, nutritionists, and physical therapists. We then divided hospitals into 3 segregation groups (low, medium, and high) based on tertiles for each of the primary diagnoses.

### Study population

For each of our study populations (primary diagnosis HF and IHD), the set of patients was limited to those admitted to hospitals for which we had a SI for that primary diagnosis. For patients with multiple admissions, only the first admission for that primary diagnosis was included. Patients with discharge dates on or after March 1, 2019, were also omitted to avoid the influence of COVID-19 on 1-year survival. We excluded patients with unknown sex (HF, n = 32; IHD, n = 41) or under 18 years of age (HF, n = 31; IHD, n = 8). We studied all medical claims associated with an admission and checked the provider type associated with the claim to ascertain whether a cardiologist saw the patient during the admission. If a claim had one of the CDM-selected clinician titles, such as cardiologist, CVD specialist, interventional cardiologist, cardiac electrophysiologist, nuclear cardiologist, or cardiac rehabilitation, it was considered that the patient was seen by a cardiologist during the admission.

### Outcomes and primary predictors

We were interested in the relationship between SI and the following outcomes: whether a patient was seen by a cardiologist, time to readmission (up to 30 days), and 1-year survival. The primary predictors of interest were segregation group (low, medium, high) and race (NH Black or NH White). For the latter 2 outcomes, we also assessed whether the patient received care from a cardiologist. As our interest is in evaluating racial disparity, results are presented in terms of Black: White odds or hazard ratios for each outcome. The source of date of death includes records from the Social Security Office and the Center for Medicare and Medicaid Services, so there is no loss-to-follow-up with 1-year survival. However, date of death was available to month only, so was assumed to be the 15th of the month for analysis of 1-year survival.

### Statistical analysis

Patient characteristics were summarized using count and percentage for categorical variables and median and quartiles for quantitative variables. For the 2 binary outcomes (whether the patient was seen by a cardiologist and 1-year survival), we used generalized linear mixed effect models. For the outcome of whether a patient was seen by a cardiologist during the admission, the model included effects of race (Black, White), segregation group (low, medium, high), interaction of race and segregation group, sex, Charlson Comorbidity Index (version including age), hospital region (Midwest, Northeast, South, West), hospital bed size (small, medium, large), and a random hospital intercept. For the outcome 1-year survival, the model also included an indicator for whether the patient was seen by a cardiologist as well as all 2-way and 3-way interactions of cardiology care, race, and segregation group.

For analysis of time-to-readmission, we excluded anyone that was not enrolled in Optum-based insurance on the date of discharge or whose enrollment ended on the day of discharge (HF, n = 816; IHD, n = 1,314) as we would have no follow-up on these patients. Patients were censored at the date their enrollment ended or 30 days, whichever came first. Because date of death was known only to the month, we could not account for this in the analysis of time-to-readmission. Analysis of time-to-readmission used a mixed-effects Cox model that included the same terms as the model for 1-year survival. Kaplan-Meier estimates were used to estimate rates of readmission at 30 days by race.

Bed size was missing for 86 of 779 hospitals (11.0%) for HF and 89 of 725 hospitals (12.3%) for IHD, so multiple imputation was used. Bed size is an ordinal variable (small, medium, large), so we used proportional odds to model this variable. Because bed size is a hospital-level variable, imputations accounted for clustering by hospital so all values for bed size within a hospital were the same for any given imputation. All analyses were conducted separately for the set of patients with a primary diagnosis of HF and for those with a primary diagnosis of IHD. For each set of patients, we also ran sensitivity analyses for subsets of patients with commercial insurance, Medicare insurance, and those with Medicare Dual (Medicare plus Medicaid) or Medicare Low Income Subsidy (LIS). Analyses were conducted in Program R version 4.1.1 and 4.3.1.^14^

## Results

### Baseline characteristics of study population

For analysis of whether the patient was seen by a cardiologist and 1-year survival, from 2010 to 2018, 119,272 patients (29.4% Black, 70.6% White) with primary diagnosis of HF and 183,165 patients (17.9% Black and 82.1% White) with primary diagnosis of IHD (Table 1) were analyzed. Among the HF cohort, a small majority of Black patients were female (55.6%), and a small majority of White patients were male (51.1%). In the IHD cohort, a small majority of Black (52.7%) patients and most White (65.6%) patients were male. In both cohorts, most Black patients (HF 58.3%, IHD 61.8%) were admitted to low segregation hospitals. For analysis of time to readmission, the populations have similar distributions (Supplemental Table 1).

**Table 1:** Baseline Characteristics of Study Populations for Seen by Cardiologist and 1-Year Survival Analyses

	Heart Failure			Ischemic Heart Disease		
	Black	White	Total	Black	White	Total
	(n = 35,038, 29.4%)	(n = 84,234, 70.6%)	(N = 119,272)	(n = 32,705, 17.9%)	(n = 150,460, 82.1%)	(N = 183,165)
Female	19,473 (55.6%)	41,157 (48.9%)	60,630 (50.8%)	15,467 (47.3%)	51,707 (34.4%)	67,174 (36.7%)
Age	72 (63-80)	77 (68-84)	75 (67-83)	68 (59-76)	69 (60-77)	69 (60-77)
Segregation						
Low	20,436 (58.3%)	32,227 (38.3%)	52,663 (44.2%)	20,210 (61.8%)	63,713 (42.3%)	83,923 (45.8%)
Medium	8,736 (24.9%)	28,758 (34.1%)	37,494 (31.4%)	7,720 (23.6%)	46,887 (31.2%)	54,607 (29.8%)
High	5,866 (16.7%)	23,249 (27.6%)	29,115 (24.4%)	4,775 (14.6%)	39,860 (26.5%)	44,635 (24.4%)
Insurance						
Commercial	4,945 (14.1%)	11,432 (13.6%)	16,377 (13.7%)	8,898 (27.2%)	49,957 (33.2%)	58,855 (32.1%)
Medicare	17,931 (51.2%)	58,277 (69.2%)	76,208 (63.9%)	15,494 (47.4%)	85,381 (56.7%)	100,875 (55.1%)
Medicare Dual	6,748 (19.3%)	7,420 (8.8%)	14,168 (11.9%)	4,203 (12.9%)	6,452 (4.3%)	10,655 (5.8%)
Medicare LIS	5,316 (15.2%)	6,806 (8.1%)	12,122 (10.2%)	4,002 (12.2%)	8,163 (5.4%)	12,165 (6.6%)
Unknown	98 (0.3%)	299 (0.4%)	397 (0.3%)	108 (0.3%)	507 (0.3%)	615 (0.3%)
Region						
Midwest	6,789 (19.4%)	24,600 (29.2%)	31,389 (26.3%)	5,951 (18.2%)	43,928 (29.2%)	49,879 (27.2%)
Northeast	2,868 (8.2%)	10,322 (12.3%)	13,190 (11.1%)	2,670 (8.2%)	16,385 (10.9%)	19,055 (10.4%)
South	24,690 (70.5%)	44,237 (52.5%)	68,927 (57.8%)	23,395 (71.5%)	80,755 (53.7%)	104,150 (56.9%)
West	691 (2.0%)	5,075 (6.0%)	5,766 (4.8%)	689 (2.1%)	9,392 (6.2%)	10,081 (5.5%)
Bed size						
Small	1993 (5.7%)	4,620 (5.5%)	6,613 (5.5%)	966 (3.0%)	5,775 (3.8%)	6,741 (3.7%)
Medium	7,742 (22.1%)	17,038 (20.2%)	24,780 (20.8%)	6,466 (19.8%)	27,949 (18.6%)	34,415 (18.8%)
Large	20,741 (59.2%)	54,068 (64.2%)	74,809 (62.7%)	20,775 (63.5%)	101,751 (67.6%)	122,526 (66.9%)
Unknown	4,562 (13.0%)	8,508 (10.1%)	13,070 (11.0%)	4,498 (13.8%)	14,985 (10.0%)	19,483 (10.6%)
Atrial fibrillation	9,609 (27.4%)	37,037 (44.0%)	46,646 (39.1%)	4,639 (14.2%)	28,831 (19.2%)	33,470 (18.3%)
COPD	13,145 (37.5%)	32,396 (38.5%)	45,541 (38.2%)	7,347 (22.5%)	31,928 (21.2%)	39,275 (21.4%)
CKD	10,704 (30.5%)	22,937 (27.2%)	33,641 (28.2%)	4,441 (13.6%)	14,869 (9.9%)	19,310 (10.5%)
Depression	1,551 (4.4%)	5,300 (6.3%)	6,851 (5.7%)	1,178 (3.6%)	6,256 (4.2%)	7,434 (4.1%)
Diabetes	18,252 (52.1%)	35,202 (41.8%)	53,454 (44.8%)	15,517 (47.4%)	55,496 (36.9%)	71,013 (38.8%)
ESRD	2,706 (7.7%)	3,470 (4.1%)	6,176 (5.2%)	1,491 (4.6%)	2,641 (1.8%)	4,132 (2.3%)
Hypertension	30,702 (87.6%)	67,717 (80.4%)	98,419 (82.5%)	28,184 (86.2%)	120,120 (79.8%)	148,304 (81.0%)
Obesity	8,530 (24.3%)	15,595 (18.5%)	24,125 (20.2%)	6,079 (18.6%)	24,952 (16.6%)	31,031 (16.9%)
Ventricular arrhythmia	2,508 (7.2%)	5,843 (6.9%)	8,351 (7.0%)	2,287 (7.0%)	11,801 (7.8%)	14,088 (7.7%)
CCI^a^	6 (5-7)	6 (5-7)	6 (5-7)	5 (3-6)	4 (3-6)	5 (3-6)

a CCI = Charlson Comorbidity Index (version including age); CKD = chronic kidney disease (stages 3-5); COPD = chronic obstructive pulmonary disease; ESRD = end-stage renal disease; LIS = low-income subsidy.

For all population analyses, Medicare was the most common insurance across race and cohort. Hypertension was the most common comorbidity across race and cohort. Atrial fibrillation was more common in White patients than Black patients in both cohorts, but particularly among those with HF. Diabetes was more common in Black patients than White patients within each cohort.

In our analysis for the HF cohort, of 779 hospitals, 260 were categorized as low segregation (range: 0.129-0.367), 259 were medium segregation (range: 0.367-0.470), and 260 were high segregation (range: 0.471-0.961). For the IHD cohort, of 725 hospitals, 242 were categorized as low segregation (range: 0.160-0.386), 241 were medium segregation (range: 0.386-0.486), and 242 were high segregation (range: 0.490-0.971).

### Care by a cardiologist

**Figure 1:**
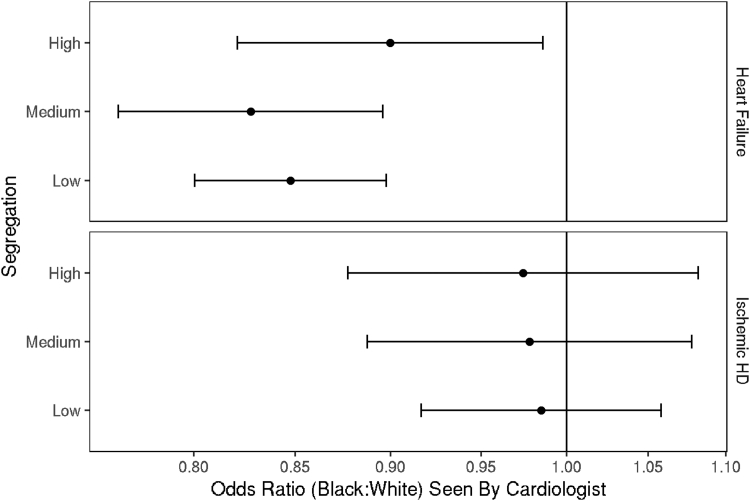
OR of Being Seen by a Cardiologist for Black Patients Compared to White Patients OR >1 indicates Blacks patients have higher odds of receipt of care by a cardiologist compared to White patients. Error bars represent 95% CIs. The model includes interactions of race and segregation group. HD = heart disease.

Within each cohort, most patients were seen by a cardiologist, with proportions similar for Black and White patients within cohort (HF: 83.2% of Black patients, 85.5% of White patients; IHD: 91.5% of Black patients, 91.4% of White patients). However, within the HF cohort, after accounting for covariates, there were significantly lower odds of being seen by a cardiologist for Black patients compared to White patients across all segregation groups (low: OR: 0.85; 95% CI: 0.80-0.90; medium: OR: 0.83; 95% CI: 0.76-0.90; high: OR: 0.90; 95% CI: 0.82-0.99 (Figure 1, Central Illustration). In contrast, within the IHD cohort, the odds of being seen by a cardiologist were similar between Black and White patients across segregation levels (Figure 1, Central Illustration).

**Central Illustration:**
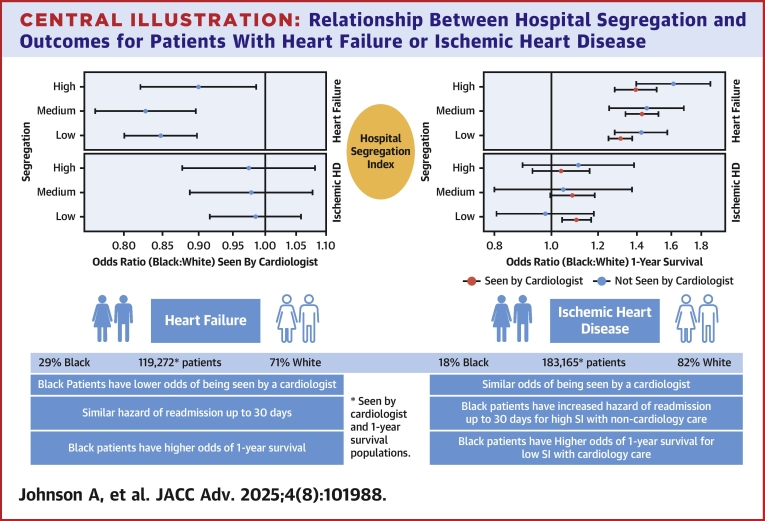
Relationship Between Hospital Segregation and Outcomes for Patients With Heart Failure or Ischemic Heart Disease Outcomes did not vary by segregation index of hospitals for patients with heart failure. Outcomes varied by segregation index for patients with ischemic heart disease when a cardiologist was included in the care. SI = segregation index; other abbreviation as in Figure 1.

### Time-to-readmission

**Figure 2:**
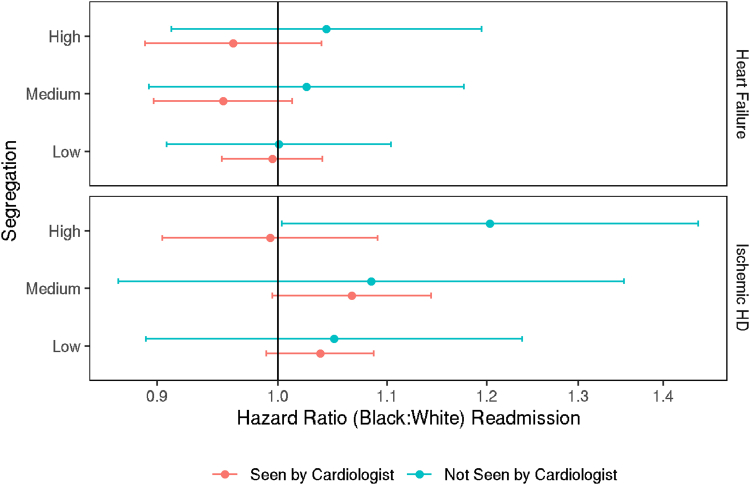
HR of Readmission by Patient Race According to Receipt of Care From a Cardiologist HR >1 indicates higher rate of readmission (up to 30 days) for Black patients compared to White patients. Error bars represent 95% CIs. The model includes all 2-way and 3-way interactions of cardiology care, race, and segregation group. Abbreviation as in Figure 1.

In the cohort of HF patients, Kaplan-Meier estimates of readmission by 30 days were similar for Black (20.8%; 95% CI: 20.4% to 21.3%) and White (21.1%; 95% CI: 20.8% to 21.4%) patients. In adjusted analysis, we found no significant difference in the hazard of readmission up to 30 days between Black and White patients across all segregation levels, whether or not they were seen by a cardiologist (Figure 2, top).

Across the IHD cohort, Kaplan-Meier estimates of readmission by 30 days were slightly higher for Black patients (14.7%; 95% CI: 14.3% to 15.1%) compared to White patients (12.7%; 95% CI: 12.6% to 12.9%). After adjusting for covariates, the hazard of readmission was not significantly different for Black patients compared to White patients for any segregation level, whether or not seen by a cardiologist, with the exception of patients not seen by cardiologist in high segregation hospitals, where the hazard of readmission was 20% higher for Black patients (HR: 1.20; 95% CI: 1.00-1.44) (Figure 2).

### One-year survival

**Figure 3:**
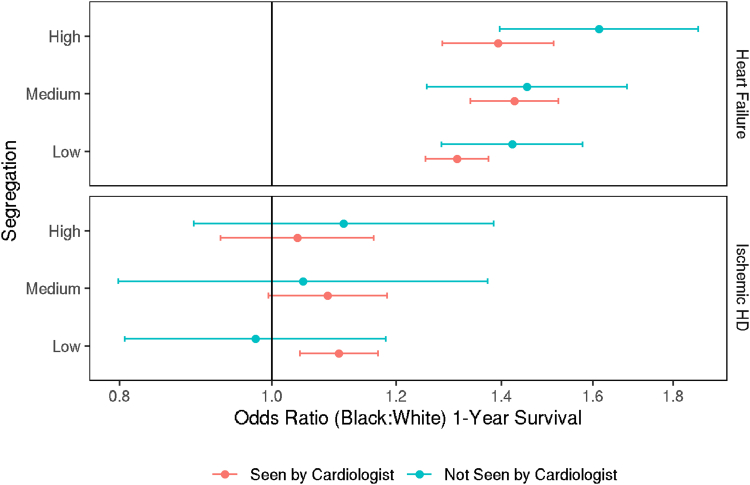
OR of 1-Year Survival by Patient Race According to Receipt of Care From a Cardiologist OR >1 indicates higher odds of 1-year survival for Black patients compared to White patients. Error bars represent 95% CIs. The model includes all 2-way and 3-way interactions of cardiology care, race, and segregation group Abbreviation as in Figure 1.

In the HF cohort, 74.7% of Black patients survived at least 1 year after discharge, compared to 67.4% of White patients. After accounting for covariates, we found significantly higher odds of 1-year survival for Black patients than White patients within each level of segregation and whether the patient received cardiology care (Figure 3, Central Illustration).

In the IHD cohort, 1-year survival rates were similar between Black (86.8%) and White (88.2%) patients. In adjusted analysis, the odds of 1-year survival for Black patients were significantly different from that of White patients only among those seen by cardiologists in low SI hospital (OR: 1.10; 95% CI: 1.04-1.17) (Figure 3, Central Illustration).

### Sensitivity analyses by insurance subsets

When stratified by insurance, odds of receipt of care by a cardiologist were similar to original findings for HF and IHD cohorts across SI tertiles (Supplemental Figure 1). For time-to-readmission (up to 30 days) and 1-year survival, the results varied for both HF and IHD cohorts with some notable observations below (Supplemental Figures 2 and 3). In the IHD cohort, Medicare beneficiaries had increased hazard of readmission for Black patients compared to White patients when receiving cardiology care at a medium-segregation hospital and when not receiving cardiology care at a high-segregation hospital. In the HF cohort, the hazard of readmission was lower for Black patients seen by a cardiologist with commercial insurance in low and high segregation hospitals and for those with Medicare Dual/LIS in medium segregation hospitals. In the HF cohort, there was a tendency for higher odds of 1-year survival for Black than White patients across most levels of segregation, cardiology care, and insurance similar to the original findings.

## Discussion

Among patients with the highest risk forms of CVD, HF, and IHD,^1^ we did not observe consistent findings between SI, receipt of care by a cardiologist, rehospitalization, and survival. Rather, we observed a complex relationship in the presence of care by a cardiologist, which was disparately lower for Black patients with HF than White patients with HF, and similar by race among patients with IHD. Outcomes did not vary by SI tertile for Black compared to White patients with HF. When including SI among patients with IHD, higher hazard of readmission was observed for Black patients at high segregation hospitals not receiving care from a cardiologist and Black patients receiving care from a cardiologist had higher odds of survival than White patients at low segregation hospitals. This suggests that more investigation is needed to understand SI and allocation to a cardiologist.

Our results are consistent with studies that have shown reduced access to cardiologists and cardiovascular therapies for Black patients with HF.^15^, ^16^, ^17^ A study revealed unequal access to cardiologists exists for Black patients with HF even in the intensive care environment.^15^ Our analysis showed Black patients had lower odds of receiving cardiology care across all hospital SI levels for the HF cohort, underpinning the complex role of hospital systems in delivering care. Many factors affect hospital care team SI, such as institutional policies; individual contributors such as implicit bias also lead to health care disparities for HF and may influence institutional policies.^8^^,^^9^^,^^18^ This anonymized study of deidentified hospitals, clinicians, and patients did not allow for examination of interpersonal racism or bias. However, clinical decisions are affected, and Black patients compared to White patients are less likely to receive advanced HF therapies, such as cardiac defibrillators, mechanical circulatory support, and heart transplantation, which lead to poorer outcomes.^5^, ^6^, ^7^^,^^9^^,^^17^^,^^19^

Our results also align with studies that have shown the importance of cardiology care compared to noncardiology care. Numerous studies have showed reduced mortality, complications, and readmissions with cardiologists compared to noncardiologists for patients with heart diseases such as HF and IHD.^20^, ^21^, ^22^ Our analysis revealed 20% higher hazard of readmission for Black patients compared to White patients when not receiving cardiology care in a high segregation hospital with IHD, further underpinning the significance of equitable access to cardiology care.

Our study differs from literature suggesting that segregated care for Black patients leads to poorer outcomes.^23^, ^24^, ^25^, ^26^ A study using Medicare beneficiaries analyzed the level of hospital segregation within the United States market using hospital referral region and dissimilarity index. They found increased acute and chronic negative outcomes for Black patients compared to White patients.^27^ In contrast, our data did not show a racially consistent trend with SI for either HF or IHD when time to readmission (up to 30 days) or 1-year survival was analyzed. Interestingly, Black patients were shown to have higher odds of 1-year survival for HF and similar levels for IHD with the exception of low segregation hospitals where survival was better for Black patients when a cardiologist was included in the care team. A potential explanation for this is in our data population. Unlike other studies, our insurance population was a mix of commercial and Medicare beneficiaries. This population has more options and selectivity for hospital teams and specialties compared to uninsured or Medicaid beneficiaries hereby modulating outcomes. There is also known discrimination toward underinsured populations,^8^^,^^28^ which may be less likely to occur with this well insured study population. However, this does not negate the possibility of mistreatment related to other patient demographics (ie, race, gender, residence, etc). Patient choice combined with access to cardiologist and geographic variability may explain some findings; a prior study using Optum data in a single state revealed better survival for Black patients with HF, IHD, or valvular heart disease when cared for by a cardiologist in hospitals with increasingly segregated clinician care by SI.^29^

Studies have shown that hospital-level effects such as risk-adjusted mortality rates, clinician practice, volume rate, and procedure rate modify levels of racial disparities in cardiovascular care.^30^^,^^31^ Significant effort is still needed to understand the role of hospital SI and its effects. Hospital care team allocation to patients is affected by numerous factors such as institutional polices, availability of specialists, implicit bias, structural racism, patient biases, and patient needs. Our study examines the degree of segregation in hospital team providers for Black and White patients and its implications for clinical outcomes. Subsequently, it highlights the need for further research. Next steps include examining hospital team and patient interactions through direct observation and focused interviews to understand nuances that contribute to patient care delivery and equity in cardiovascular care.^32^

### Study limitations

First, we acknowledge that Optum’s deidentified CDM does not include all patients seen by a single provider, and there could be different segregation indices when a total panel is accounted for. Second, patient IHD was not categorized into risk level (medical management vs coronary revascularization procedure), and neither was HF class or type known. Third, hospitals participating in national quality improvement projects such as Get with the Guidelines-Heart Failure were not identified, which could have contributed to our analysis as it has been shown to reduce disparities across race and insurance.^33^ Finally, hospital teams were determined using a claims-based approach, so only individual providers who billed during patient’s hospital stay as provider category as cardiologist were included.

## Conclusions

Among Black and White patients with commercial and Medicare insurance, there was significantly reduced access to cardiology care for Black patients with HF. SI did not demonstrate a consistent trend for time to readmission (up to 30 days) and 1-year survival for both HF and IHD cohorts. Insurance type may have moderated outcomes; hence, further research is needed to investigate hospital-level factors such as hospital care team selection, availability, and receipt of cardiovascular care by patients.

Perspectives **COMPETENCY IN PATIENT CARE:** Black patients with HF have reduced access to cardiologists. More work is needed to close the care gap. This was not an issue for patients with IHD.

**COMPETENCY IN SYSTEMS-BASED PRACTICE:** Hospital systems play a complex role in delivering health care. In a population of adequately insured patients, hospital team segregation did not have a consistent relationship to rehospitalization or mortality. Patterns varied with the receipt of cardiovascular care.

**TRANSLATIONAL OUTLOOK:** Racial disparities persist. Hospital-level factors that contribute to inequitable cardiovascular care and outcomes require further granular investigation. Direct observation with qualitative methods may be enlightening.

## Funding support and author disclosures

This study was funded by Dr Breathett’s research support from the 10.13039/100000102Health Resources and Services Administration (HRSA) of the 10.13039/100000016U.S. Department of Health and Human Services (HHS). This research was also supported in part by Lilly Endowment, Inc, through its support for the Indiana University Pervasive Technology Institute. Dr Breathett also receives research funding from the 10.13039/100000050National Heart, Lung, and Blood Institute (NHLBI) R01HL159216. The contents are those of the author(s) and do not necessarily represent the official views of, nor an endorsement, by Lilly, HRSA, HHS, or the U.S. Government. The authors have reported that they have no relationships relevant to the contents of this paper to disclose.
