# Supplementary material for: Relationship Between Hospital Team Segregation Index, Heart Failure, and Ischemic Heart Disease
Source: JACC Adv. 2025 Jul 18;4(8):101988. doi: 10.1016/j.jacadv.2025.101988 (PMC12301724; doi:10.1016/j.jacadv.2025.101988)
Supplement: Supplementary Material [file mmc1.docx]

**SUPPLEMENTAL APPENDIX**

***Sensitivity Analyses by Insurance Subsets***

We examined our data by insurance subsets. For HF cohort, Black patients had statistically significant lower odds of being seen by a cardiologist compared to White patients for commercial insurance (low and high segregation group) and Medicare insurance (low and medium segregation group) (**Supplemental Figure 1, left**). In contrast IHD patients did not show any significant difference across segregation groups and in any of the insurance subsets (**Supplemental Figure 1, right**).

For time to readmission (up to 30 days), HF cohort did not show a significant difference across race when stratified using insurance except for commercial beneficiaries when seen by a cardiologist at a low segregation hospital (HR 0.84, 95% CI 0.72-0.99) and high segregation hospital (HR 0.82, 95% Cl 0.67-0.99). Medicare Dual/LIS insurance beneficiaries when seen by a cardiologist at a medium segregation hospital (OR 0.88, 95% CI 0.79-0.98) (**Supplemental Figure 2, left**). Similarly, in IHD cohort there were no significant differences in hazard of readmission by race when stratifying by insurance with the exception of patients on Medicare for whom the hazard of readmission was significantly higher for Black patients among those seen by a cardiologist at a medium segregation hospital (HR 1.13, 95%CI 1.03-1.24) and among those not seen by a cardiologist at a high segregation hospital (HR 1.33. 1.07-1.66; **Supplemental Figure 2, right**).

For one year survival post discharge, there was a tendency for higher odds of 1-year survival for Black than White patients across all segregation groups, whether or not seen by a cardiologist and insurance types for HF cohort. However, the effect was not statistically significant in medium segregation group for commercial beneficiaries not seen by a cardiologist (**Supplemental Figure 3, left**). For IHD cohort, Medicare and Medicare Dual/LIS beneficiaries had significant higher odds of 1-year survival for Black patients than White patients in low segregation (OR 1.16, 95% CI 1.07 – 1.24) and high segregation (OR 1.35, 95% CI 1.06 – 1.71) respectively when seen by a cardiologist. Among patients not seen by a cardiologist in high SI hospitals within the Medicare Dual/LIS subgroup, Black patients had higher odds of 1-year survival than White patients (OR 1.66 95% CI 1.01-2.72 ) (**Supplemental Figure 3, right**).

|  | Heart Failure | | |  | Ischemic Heart Disease | | |
| --- | --- | --- | --- | --- | --- | --- | --- |
|  | Black | White | Total |  | Black | White | Total |
|  | 34,796 (29.4 %) | 83,660 (70.6%) | 118,456 |  | 32,445 (17.8%) | 149,406 (82.2%) | 181,851 |
| Male | 15,442 (44.4%) | 42,758 (51.1%) | 58,200 (49.1%) |  | 17,092 (52.7%) | 98,043 (65.6%) | 115,135 (63.3%) |
| Female | 19,354 (55.6%) | 40,902 (48.9%) | 60,256 (50.9%) |  | 15,353 (47.3%) | 51,363 (34.4%) | 66,716 (36.7%) |
| Segregation |  |  |  |  |  |  |  |
| Low | 20,323 (58.4%) | 32,018 (38.3%) | 52,341 (44.2%) |  | 20,048 (61.8%) | 63,294 (42.4%) | 83,342 (45.8%) |
| Medium | 8,661 (24.9%) | 28,547 (34.1%) | 37,208 (31.4%) |  | 7,657 (23.6%) | 46,532 (31.1%) | 54,189 (29.8%) |
| High | 5,812 (16.7%) | 23,095 (27.6%) | 28,907 (24.4%) |  | 4,740 (14.6%) | 39,580 (26.5%) | 44,320 (24.4%) |
| Insurance |  |  |  |  |  |  |  |
| Commercial | 4,904 (14.1%) | 11,286 (13.5%) | 16,190 (13.7%) |  | 8,827 (27.2%) | 49,544 (33.2%) | 58,371 (32.1%) |
| Medicare | 17,864 (51.3%) | 58,056 (69.4%) | 75,920 (64.1%) |  | 15,419 (47.5%) | 85,117 (57.0%) | 100,536 (55.3%) |
| Medicare Dual | 6,707 (19.3%) | 7,377 (8.8%) | 14,084 (11.9%) |  | 4,186 (12.9%) | 6,414 (4.3%) | 10,600 (5.8%) |
| Medicare LIS | 5,292 (15.2%) | 6,786 (8.1%) | 12,078 (10.2%) |  | 3,985 (12.3%) | 8,133 (5.4%) | 12,118 (6.7%) |
| Unknown | 29 (0.1%) | 155 (0.2%) | 184 (0.2%) |  | 28 (0.1%) | 198 (0.1%) | 226 (0.1%) |
| Region |  |  |  |  |  |  |  |
| Midwest | 6,741 (19.4%) | 24,441 (29.2%) | 31,182 (26.3%) |  | 5,904 (18.2%) | 43,580 (29.2%) | 49,484 (27.2%) |
| Northeast | 2,846 (8.2%) | 10,249 (12.3%) | 13,095 (11.1%) |  | 2,649 (8.2%) | 16,270 (10.9%) | 18,919 (10.4%) |
| South | 24,520 (70.5%) | 43,915 (52.5%) | 68,435 (57.8%) |  | 23,204 (71.5%) | 80,209 (53.7%) | 103,413 (56.9%) |
| West | 689 (2.0%) | 5,055 (6.0%) | 5,744 (4.8%) |  | 688 (2.1%) | 9,347 (6.3%) | 10,035 (5.5%) |
| Bed Size |  |  |  |  |  |  |  |
| Small | 1,979 (5.7%) | 4,594 (5.5%) | 6,573 (5.5%) |  | 964 (3.0%) | 5,741 (3.8%) | 6,705 (3.7%) |
| Medium | 7,684 (22.1%) | 16,943 (20.3%) | 24,627 (20.8%) |  | 6,408 (19.8%) | 27,750 (18.6%) | 34,158 (18.8%) |
| Large | 20,603 (59.2%) | 53,675 (64.2%) | 74,278 (62.7%) |  | 20,609 (63.5%) | 101,033 (67.6%) | 121,642 (66.9%) |
| Unknown | 4,530 (13.0%) | 8,448 (10.1%) | 12,978 (11.0%) |  | 4,464 (13.8%) | 14,882 (10.0%) | 19,346 (10.6%) |
| Atrial Fibrillation | 9,564 (27.5%) | 36,808 (44.0%) | 46,372 (39.1%) |  | 4,597 (14.2%) | 28,632 (19.2%) | 33,229 (18.3%) |
| COPD | 13,068 (37.6%) | 32,203 (38.5%) | 45,271 (38.2%) |  | 7,286 (22.5%) | 31,729 (21.2%) | 39,015 (21.5%) |
| CKD | 10,634 (30.6%) | 22,795 (27.2%) | 33,429 (28.2%) |  | 4,418 (13.6%) | 14,758 (9.9%) | 19,176 (10.5%) |
| Depression | 1,543 (4.4%) | 5,276 (6.3%) | 6,819 (5.8%) |  | 1,173 (3.6%) | 6,214 (4.2%) | 7,387 (4.1%) |
| Diabetes | 18,145 (52.1%) | 34,995 (41.8%) | 53,140 (44.9%) |  | 15,426 (47.5%) | 55,139 (36.9%) | 70,565 (38.8%) |
| ESRD | 2,692 (7.7%) | 3,434 (4.1%) | 6,126 (5.2%) |  | 1,478 (4.6%) | 2,599 (1.7%) | 4,077 (2.2%) |
| Hypertension | 30,528 (87.7%) | 67,328 (80.5%) | 97,856 (82.6%) |  | 28,018 (86.4%) | 119,472 (80.0%) | 147,490 (81.1%) |
| Obesity | 8,474 (24.4%) | 15,498 (18.5%) | 23,972 (20.2%) |  | 6,038 (18.6%) | 24,799 (16.6%) | 30,837 (17.0%) |
| Ventricular Arrythmia | 2,479 (7.1%) | 5,787 (6.9%) | 8,266 (7.0%) |  | 2,239 (6.9%) | 11,606 (7.8%) | 13,845 (7.6%) |

**Supplemental Table 1. Baseline Characteristics of Study Populations for Time-to-Readmission Analyses**

*CCI, Charlson Comorbidity Index (version including age); COPD: Chronic Obstructive Pulmonary Disease; CKD: Chronic Kidney Disease (stages 3-5); ESRD: End Stage Renal Disease; LIS: low income subsidy

**Supplemental Figure 1. Odds ratio of being seen by a cardiologist for Black patients compared to White patients across segregation levels and insurance types for HF and IHD**

**
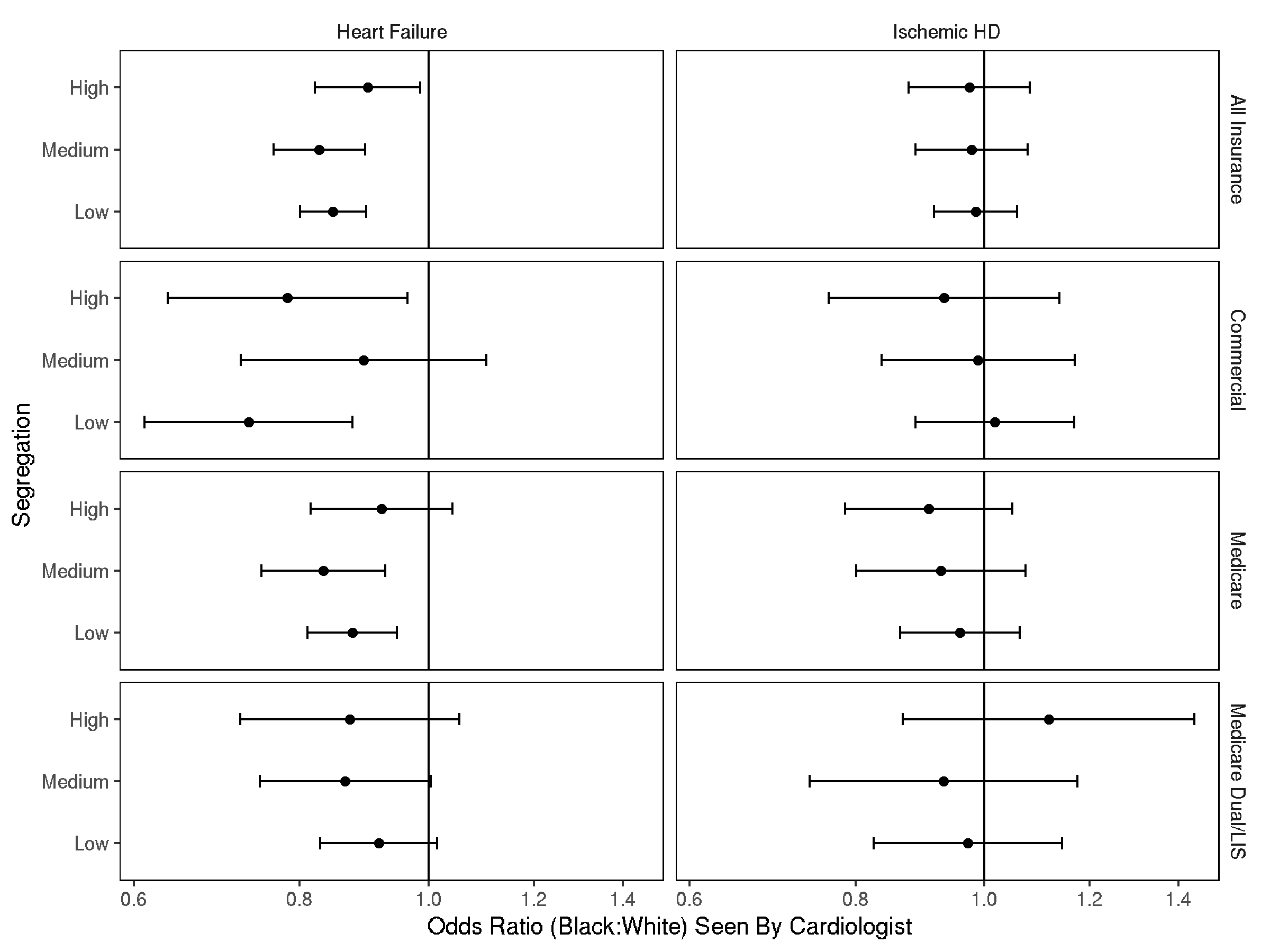
**

Odds ratio greater than 1 indicates Blacks patients have higher odds of receipt of care by a cardiologist compared to White patients. Error bars represent 95% Confidence Intervals. The models include all 2-way and 3-way interactions of cardiology care, race, and segregation group.

**Supplemental Figure 2. Hazard ratio of readmission for Black patients compared to White patients across segregation levels and insurance types for HF and IHD according to receipt of care by a cardiologist**


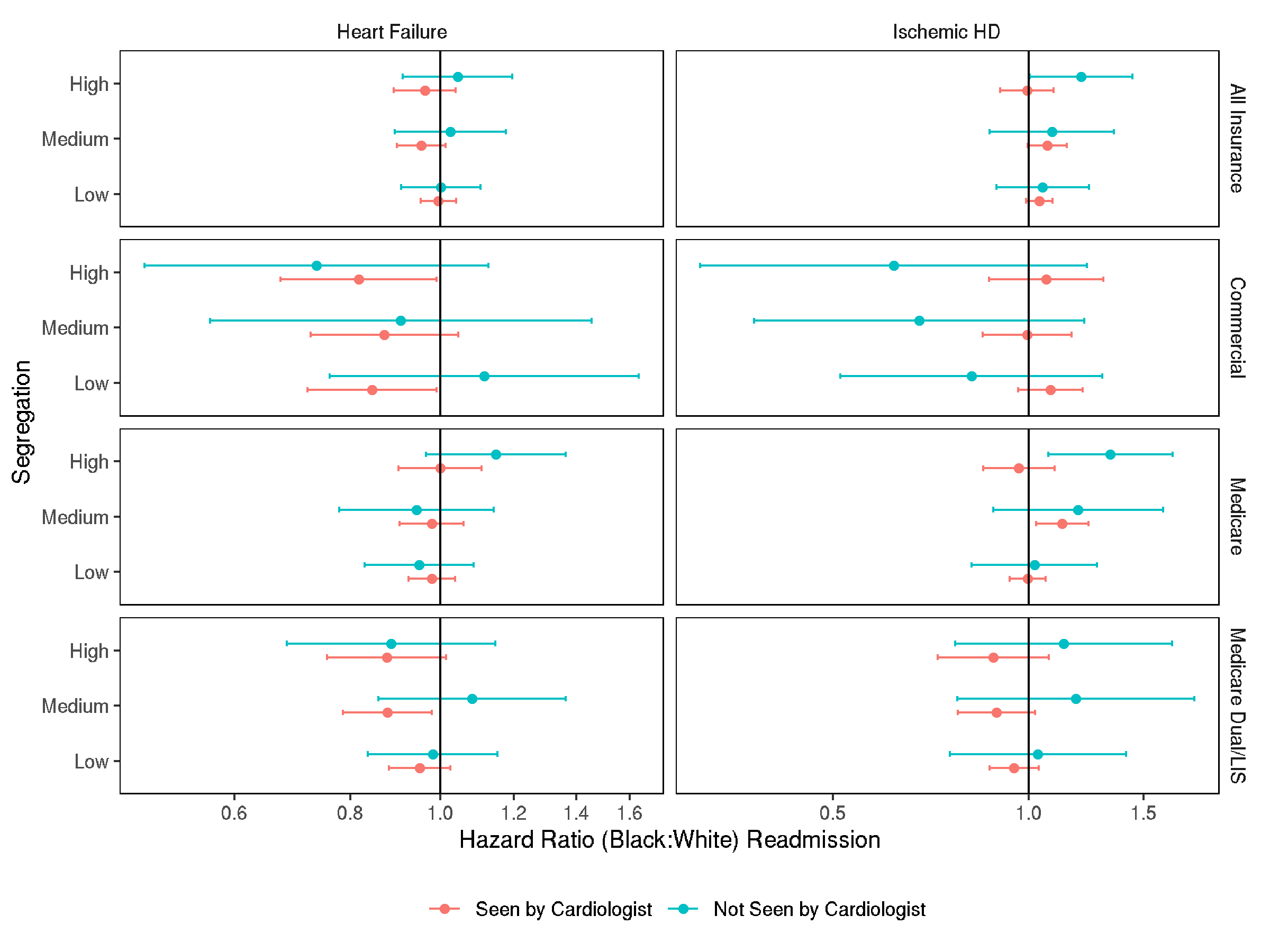


Hazard ratio greater than 1 indicates higher hazard of readmission in the first 30-days after discharge for Black patients compared to White patients. Error bars represent 95% Confidence Intervals. The models include whether the patient was seen by a cardiologist as well as all 2-way and 3-way interactions of cardiology care, race, and segregation group.

**Supplemental Figure 3. Odds ratio of 1-year survival for Black patients compared to White patients across segregation levels and insurance levels for HF and IHD according to receipt of care by a cardiologist**

**
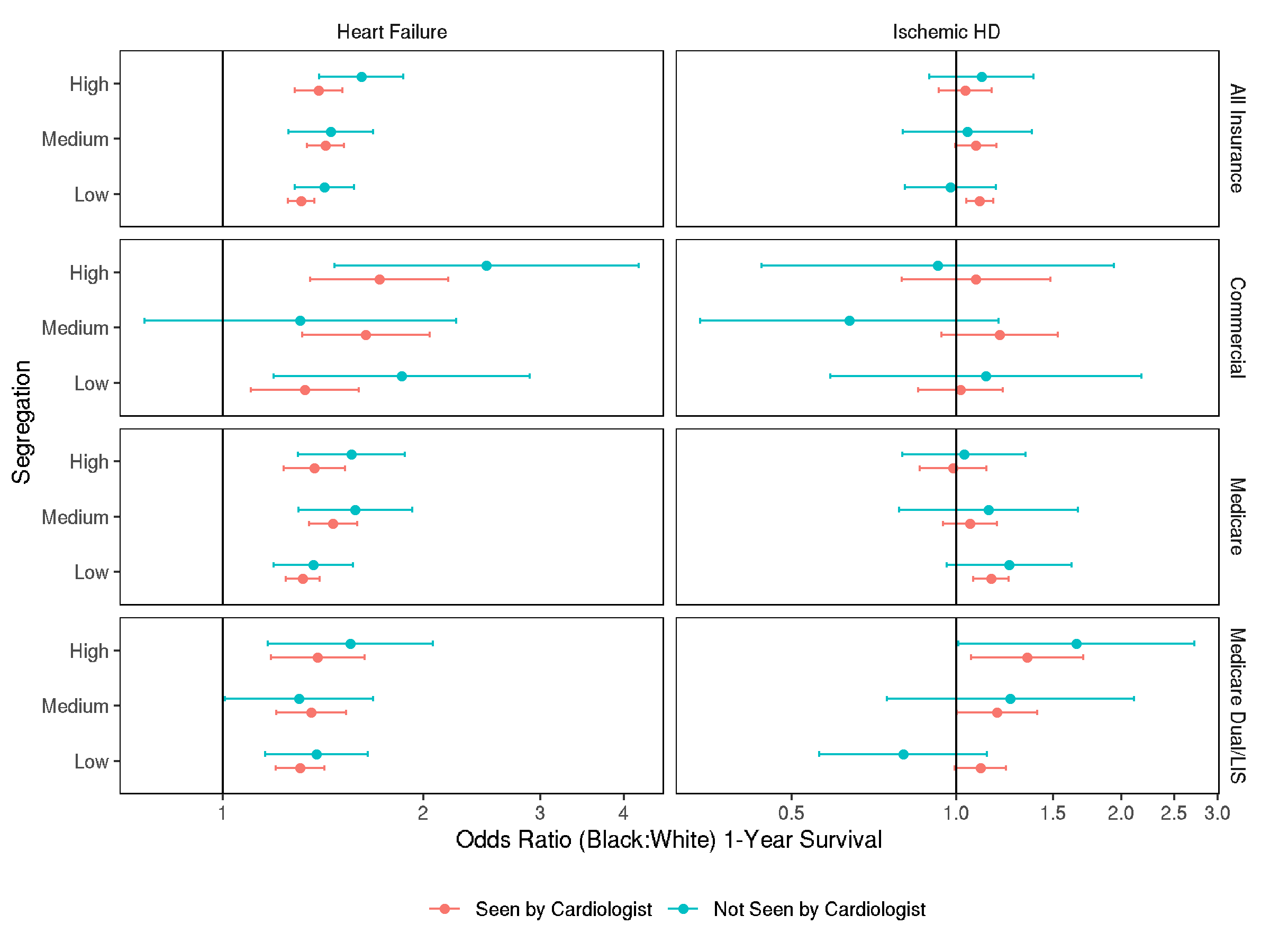
**

Odds ratio greater than 1 indicates higher odds of 1-year survival for Black patients compared to White patients. Error bars represent 95% Confidence Intervals. The models include whether the patient was seen by a cardiologist as well as all 2-way and 3-way interactions of cardiology care, race, and segregation group.
